# Use of ADHD Medication Among Danish Children and Adolescents from 2010-2020 - A Nationwide Study

**DOI:** 10.1192/j.eurpsy.2022.249

**Published:** 2022-09-01

**Authors:** M. Stoltz-Andersen, R. Wesselhoeft, M. Ernst, L. Rasmussen

**Affiliations:** University of Southern Denmark, Ist - Clinical Pharmacology, Pharmacy And Environmental Medicine, Odense M, Denmark

**Keywords:** children and adolescent, drug utilization, nationwide, adhd

## Abstract

**Introduction:**

To ensure rational drug use, there is a need to continuously monitor the use of ADHD medication among children and adolescents.

**Objectives:**

To describe the use of ADHD medication among Danish children and adolescents from 2010-2020.

**Methods:**

Using the Danish national healthcare registries, we extracted data on filled prescriptions of ADHD medication (including methylphenidate, atomoxetine, guanfacine, dexamphetamine, and lisdexamphetamine) among children (age 6-12 years) and adolescents (age 13-17 years) between 2010-2020. We examined the annual incidence rate and prevalence proportion of ADHD drug use, and the proportion of children and adolescents having an ADHD diagnosis when initiating ADHD medication.

**Results:**

From 2010-2020, the incidence followed a u-shaped trend with an incidence rate of 4.9/1,000 children and 4.4/1,000 adolescents in 2010, decreasing to 3.2/1,000 children and 3.0/1,000 adolescents in 2013, and rising to 4.9/1,000 children and 4.8/1,000 adolescents in 2020. The prevalence for children showed a similar trend, shifting from 17/1,000 in 2010, to 15/1,000 in 2016, and peaking at 19/1,000 children in 2020. However, among adolescents the prevalence increased steadily from 19/1,000 in 2010 to 29/1,000 in 2020. 67% of children and 53% of adolescents initiating ADHD medication had an ADHD diagnosis.

**Conclusions:**

After an initial decline in incidence rates of ADHD medication use among Danish children and adolescents, there has been a rise in use the last five years. The same trend applied for the prevalence among children, whereas the prevalence among adolescents increased steadily over the entire period. More than half of children and adolescents initiating ADHD medication were diagnosed with ADHD.

**Disclosure:**

No significant relationships.

